# Web-Based Multifaceted Approach for Community-Based HIV Self-Testing Among Female Sex Workers in Indonesia: Protocol for a Randomized Community Trial

**DOI:** 10.2196/27168

**Published:** 2021-07-21

**Authors:** Jessie Olivia Yunus, Anak Agung Sagung Sawitri, Dewa Nyoman Wirawan, I Gusti Agung Agus Mahendra, Dewi Susanti, Ni Kadek Ayu Dwi Utami Ds, Dedison Asanab, Ida Ayu Narayani, Oldri Sherli Mukuan, Asti Widihastuti, Robert Magnani, Pande Putu Januraga

**Affiliations:** 1 Kerti Praja Foundation Denpasar Bali Indonesia; 2 Department of Public Health and Preventive Medicine Faculty of Medicine Udayana University Denpasar Bali Indonesia; 3 United Nations Population Fund Indonesia Jakarta Indonesia; 4 Faculty of Public Health University of Indonesia Depok Indonesia

**Keywords:** HIV, self-testing, oral fluid test, community-based screening, female sex worker, Indonesia

## Abstract

**Background:**

New HIV infections in Indonesia continue to be concentrated among key populations, including female sex workers (FSWs). However, increasing HIV testing among this subpopulation remains a challenge, necessitating exploration into alternative testing modalities.

**Objective:**

This study aims to assess whether the addition of an oral fluid testing option in community settings would increase the rate of HIV case identification among FSWs. Because the study was implemented early in the outbreak of COVID-19 in Indonesia, a secondary objective is to assess approaches and tools for implementing both community outreach and community HIV screening for FSWs during pandemic conditions.

**Methods:**

We undertook a community-based randomized trial in 23 national priority districts in which community outreach services were being provided. Community-based screening using an oral fluid-based rapid test was added to the community outreach standard of care in intervention districts with clients having the option of performing the test themselves or being assisted by outreach workers. A web-based system was created to screen for eligibility and collect participant data and test results, facilitating the process for both unassisted and assisted participants. Participants with reactive screening results were encouraged to undergo HIV testing at a health facility to confirm their diagnosis and initiate antiretroviral treatment as needed. Multiple means of recruitment were deployed including through outreach workers and social media campaigns.

**Results:**

Of the 1907 FSWs who registered, met the eligibility criteria, and gave consent to participate, 1545 undertook community oral fluid test (OFT) screening. Most (1516/1545, 98.1%) opted for assisted screening. Recruitment via social media fell far short of expectations as many who registered independently for the OFT because of the social media campaign did not identify as FSWs. They were eventually not eligible to participate, but their interest points to the possibility of implementing HIV self-testing in the general population. The successful recruitment through outreach workers, facilitated by social media, indicates that their roles remain crucial in accessing FSW networks and improving HIV testing uptake.

**Conclusions:**

The addition of HIV self-testing to the standard of care supported by a web-based data collection system was able to increase HIV case identification among FSWs in intervention districts. The high satisfaction of OFT users and the interest of the general population toward this alternative testing modality are promising for scaling up community HIV screening nationally.

**Trial Registration:**

ClinicalTrials.gov NCT04578145; https://clinicaltrials.gov/ct2/show/NCT04578145

**International Registered Report Identifier (IRRID):**

RR1-10.2196/27168

## Introduction

### HIV Epidemic in Indonesia

Indonesia is one of the countries with high HIV burden in World Health Organization’s (WHO) South-East Asian Region, having 20% of the region’s people living with HIV and being the only high burden country estimated to experience continued increases in people living with HIV [1] and AIDS-related death [2]. Although overall HIV prevalence among most population subgroups is projected to decline between 2019 and 2024, the number of AIDS-related deaths among these populations is projected to increase [3]. It has been estimated that only 51% of people living with HIV in Indonesia know their status [2], and only 23% of adult people living with HIV have been estimated to be on antiretroviral therapy (ART) [3], making Indonesia one of the countries falling behind in reaching the 90-90-90 goal. However, with commitments to increase ART coverage in the latest National Strategic Plan, ART coverage is targeted to reach at least 75% by 2024 [4] to reverse the trend of AIDS-related deaths [3].

New HIV infections in Indonesia are concentrated among key population groups, including female sex workers (FSWs). Sex work was previously categorized as “direct” (ie, based in brothels, *lokalisasi*, streets, etc) and “indirect” (ie, based in entertainment facilities, such as bars and karaoke clubs, massage parlors, etc), and it is believed that many FSWs have shifted their work to indirect due to policies mandating the closing of brothels [3,5]. The term *lokalisasi* is an Indonesian word meaning “localization,” which describes areas designated for sex work establishments. FSWs have also become harder to reach, as previous prevention efforts were based on established locations of sex work activities and as digital platforms have gained popularity to facilitate transactions [5].

In 2019, it was estimated that there were 277,624 FSWs throughout Indonesia with around 4,688,216 clients annually [3]. This mode of transmission continues to the clients’ other sexual partners and their children. Lowering HIV transmission between FSWs and their clients will lower onward transmission. To increase ART coverage, more undiagnosed people living with HIV need to know their status. Given the increasingly challenging landscape in sex work in Indonesia, alternative strategies for increasing HIV testing uptake among FSWs are needed [6].

### HIV Testing Among FSWs

The FSW population is at greater risk for not only HIV infection, but also stigma, discrimination, and violence due to the nature of sex work, which can affect their access to HIV testing [7]. Achieving high HIV testing coverage among FSWs is a global challenge [8]. While there is sizeable literature on the efficacy of community-based HIV prevention interventions for FSWs [9-12], there is limited global evidence on how to best increase rates of HIV testing among FSWs. Although the WHO [13] strongly recommended community-based testing approaches to supplement facility-based HIV testing, available evidence on the efficacy of this approach among FSWs is quite limited. A recent systematic review [8] reported 10 studies that examined recent testing in response to HIV promotion interventions for FSWs. Reported HIV testing uptake was found to vary, for example, in Canada, it was reported that 76% FSWs had HIV tests within the last 1 year [14], while in China, only 22% FSWs reported taking HIV tests within the last 1 year [15]. Reported barriers for service access included financial factors, time, stigma, discrimination, low-risk perception, fear, lack of accessibility, reluctance from health service providers to offer HIV testing, and limited human resources. A review by Tokar et al also found that social support from peers and managers could increase HIV testing uptake among FSWs [8].

### Community-Based HIV Screening

At present, the only HIV testing performed outside of health facilities in Indonesia is via mobile clinics deployed from government community health centers. Although this eliminates the need to go to health facilities for testing, the linkage of testing with treatment has proven problematic for the mobile clinic strategy. The cost-effectiveness of this approach, at least in Jakarta, has also been questioned [6]. The cost of mobile voluntary counselling and testing to identify one HIV positive case among FSWs was almost six times higher than that to identify one HIV positive case among transgender individuals and men who have sex with men (MSM) and 17 times higher than that to identify one HIV positive case among people who inject drugs.

The latest WHO HIV testing guidelines [16] highlight community-based and HIV self-testing (HIVST) as a tool to identify more people with undiagnosed HIV who are at high risk of HIV infection. Privacy, confidentiality, and elimination of stigma are some of the advantages offered by this method in removing barriers to service access. UNAIDS [7] also suggested that HIVST has the potential to increase access to HIV testing especially among people living with HIV and key population groups who do not yet know their status. Although the acceptability of HIVST, which includes both oral fluid test (OFT) and blood-based kits, has been studied among key populations, the studies are overwhelmingly performed among MSM in high-income countries and with varied types of supervisions [17-22]. However, the studies performed among FSWs demonstrated HIVST as a highly accepted strategy [21,23-26], which is a promising alternative to facility-based HIV testing.

In Indonesia, there have been few studies on the use of the OFT among key populations. One pilot study conducted among MSM used the OFT in exploring alternatives to facility-based HIV testing [27]. Although more MSM were able to know their status, this study found that only 38% of MSM with reactive OFT results went to health facilities for a confirmatory test, which is a requirement to receive government-subsidized HIV treatment. Another study, the HIV Awal (Early) Testing & Treatment Indonesia (HATI) Study [28], explored the impact of different approaches to HIV care, including use of the OFT among MSM, FSWs, *waria* (transgender women), and people who inject drugs. However, participants recruited were mostly MSM, and there was considerable difficulty recruiting FSWs at two of the three implementation sites [29].

An approach that has been increasingly employed to improve HIV testing uptake is internet-based outreach, which utilizes social networking sites [30], mobile apps [31], and websites [32]. The benefit of internet recruitment as opposed to traditional in-person recruitment is its ability to reach participants beyond the local area [33]. Furthermore, trials utilizing the web-based approach can also be combined with self-testing using the OFT, allowing the entire counselling and testing process to be conducted remotely [34,35]. This approach not only allows users to test at their convenience, but also allows testing and data collection to be performed in private, supporting HIV testing uptake [36]. However, a recent systematic review on technology-supported HIV testing revealed that most of these trials were performed among MSM, with only one trial performed among FSWs utilizing text messaging instead of an internet-based approach [36]. In light of this, it is essential to examine how implementing community-based HIVST supported with the internet will impact HIV testing uptake among FSWs in Indonesia.

The HIVST strategy using the OFT is yet to be employed to increase testing among FSWs in Indonesia, a key population group that has become increasingly hidden due to persistent police harassment [37] and brothel closures. Accordingly, a randomized community trial was designed to examine whether shifting screening from health facilities to community settings using noninvasive HIVST with the OFT can increase the number of FSWs who know their HIV status and subsequently increase treatment uptake for those who test positive. The acceptability and potential differences between assisted and unassisted testing can also be evaluated, as was observed in the study by Nguyen et al [25], where the cases were lower among participants undergoing lay-provider testing as opposed to self-testing.

### Aim

This trial aims to answer the primary and secondary objectives outlined below. The initial trial protocol was designed prior to the COVID-19 pandemic. Although data collection websites were part of the initial plan, support activities, such as training, liaising, data monitoring, and participant recruitment, were designed to be carried out in person. This paper aims to describe the study protocol that was configured such that it could be implemented during COVID-19 pandemic conditions through internet and telecommunication technologies.

The primary objective of the study is to answer the following questions: (1) Does the introduction of community-based HIV screening (CBS) increase the proportion of HIV cases confirmed through facility-based testing and subsequent linkage to care among FSWs? (2) Are there differences in the proportion of cases confirmed through facility-based testing and subsequent linkage to care among FSWs undergoing assisted and unassisted CBS?

The secondary research questions are as follows: (1) How is CBS perceived among FSWs? (2) What are the differences in the characteristics of FSWs receiving assisted and unassisted CBS?

## Methods

### Study Setting

**Figure 1:**
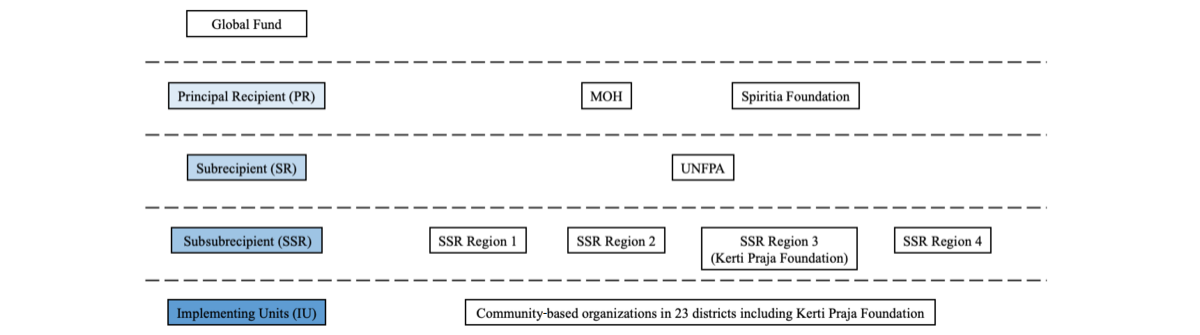
HIV program network in Indonesia. MoH: Ministry of Health; UNFPA: United Nations Population Fund.

This trial is integrated into the structure of HIV prevention programs in Indonesia, which consists of different levels of implementors and networks (Figure 1). HIV prevention and treatment have long involved FSW peer leaders and non-FSW outreach workers (OWs), and Kerti Praja Foundation is an implementing unit for HIV programs and an implementing partner for this study. All peer leaders and non-FSW OWs will be collectively referred as OWs. Following brothel closures in 2016, United Nations Population Fund (UNFPA) Indonesia, the funding agency for this study, initiated a unique programmatic change that aims to have a minimum of 80% of OWs who are current or former sex workers. This was done to strengthen outreach as previous structures of sex work became fragmented [5] and have not previously been implemented by other large-scale programs where OWs are familiar with the FSW network, but might not be FSWs themselves. Furthermore, a national network of sex workers, *Organisasi Perubahan Sosial Indonesia* (OPSI) or Organisation of Social Change in Indonesia, had an advisory role from the early stages of this study during proposal development and remained closely engaged throughout study implementation. As such, the instruments and manual of the operating procedure of the study were developed and revised in consultation with FSWs and OWs.

Outreach activities by OWs were performed through both face-to-face and virtual methods, with the latter being an alternative when physical contact was too risky for OWs and FSWs. An infection control protocol was developed by the United Nations Population Fund (as per unpublished 2020 report "Panduan Bagi Peer Leader dalam Situasi COVID-19" by the United Nations Population Fund, Jakarta, Indonesia) for OWs when face-to-face outreach was necessary.

Sustained engagement with key stakeholders gave rise to an evaluative qualitative study, which was developed and led by researchers from OPSI. FSWs were purposively selected from the CBS trial to reflect insights from a diversity of participants. Details of the evaluative study will not be covered in this article, as it aims to describe the protocol of the main trial.

### Study Design

**Figure 2:**
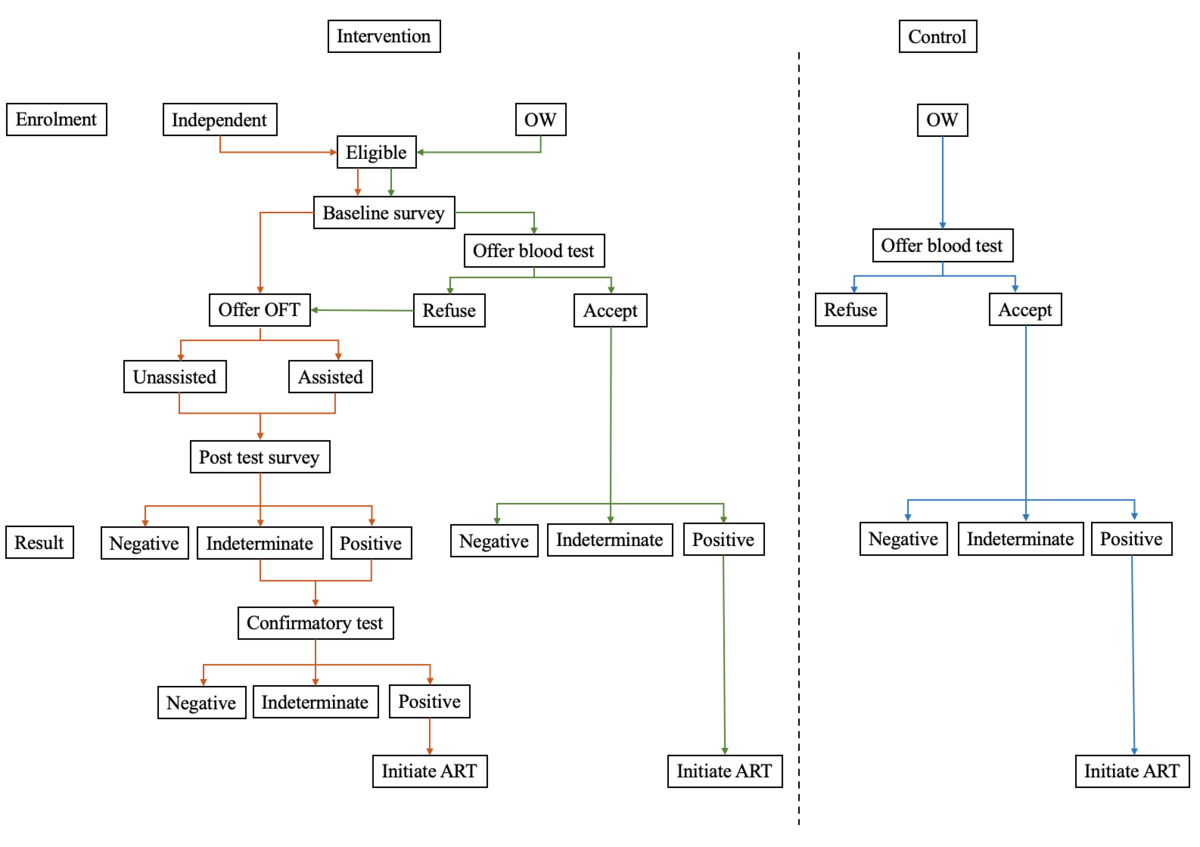
Study flowchart outlining processes in the intervention and control arms. ART: antiretroviral therapy; OFT: oral fluid test; OW: outreach worker.

The design of this study is a randomized community trial whereby priority districts are randomized into intervention and control arms [38]. Control districts implemented the community outreach standard of care. The current standard HIV prevention program involves implementing units, which are community-based organizations that coordinate outreach to FSWs. Outreach activities include distribution of educational media on HIV and AIDS, distribution of condoms and lubricants, and advocacy for FSWs to receive periodic HIV tests at health care facilities or through mobile clinics deployed by the Ministry of Health. Each implementing unit was given a target number of HIV tests that renews every 6 months. FSWs with positive test results are referred to initiate ART (Figure 2).

In the intervention districts, in addition to standard of care, FSWs were reached through multiple methods. Those recruited through OWs were given the option of choosing the OFT if they refused facility-based HIV blood tests. Conversely, FSWs recruited independently through social media could only select the OFT. All participants choosing the OFT could select either the assisted or unassisted method. Study kits were delivered through OWs for the assisted method and through courier services for the unassisted method. Participants with indeterminate or reactive OFT results were encouraged to access health services for confirmatory blood testing and proceed to initiate ART if confirmed to be HIV positive. All participants in the intervention arm completed baseline surveys, while only OFT participants completed posttest surveys. After uploaded data were verified, both participants and OWs were compensated in Indonesian Rupiah (IDR) as indicated in Table 1.

**Table 1:** Incentives for outreach workers and female sex workers.

Arm		Incentive (IDR)^a^	
		Outreach workers	Female sex workers
**Control (standard of care)**			
	Facility-based testing	70,000	N/A^b^
	Mobile VCT^c^	25,000	N/A
**Intervention**			N/A
	Facility-based testing	70,000	N/A
	Mobile VCT	25,000	N/A
	Unassisted OFT^d^	100,000	N/A
	Assisted OFT	150,000	N/A
	Baseline survey	N/A	50,000
	HIV test (blood test, OFT, confirmatory test)	N/A	100,000
	Posttest survey	N/A	50,000

^a^The average Indonesian Rupiah (IDR)/USD exchange rate during the study period was USD 1 = IDR 14,600.

^b^N/A: not applicable.

^c^VCT: voluntary counselling and testing.

^d^OFT: oral fluid test.

### District Allocation

The districts in the study consisted of the 23 cities and regencies designated by the Indonesian Ministry of Health as being the highest priority for HIV and AIDS programming. Allocation to study arms was performed using stratified randomization by firstly creating sampling strata through sorting from largest to smallest the mean average of achieved HIV testing per semester from 2018 to 2019 in each district. The 23 districts were then sorted into seven groups of three districts and one group of two districts. Randomization for the intervention and comparison groups was performed with a ratio of 2:1 to increase the likelihood of reaching the target number of FSWs receiving the OFT. The districts serving as intervention sites included Medan City, Deli Serdang Regency, Palembang City, Tangerang Selatan City, Tangerang Regency, East Jakarta City, Central Jakarta City, West Jakarta City, Bogor Regency, Depok City, Surakarta City, Malang City, Surabaya City, Denpasar City, and Sorong City. The districts serving as control sites included Bandar Lampung City, South Jakarta City, North Jakarta City, Bandung City, Depok City, Semarang City, Makassar City, and Jayapura City.

### Study Population

This trial aims to recruit FSWs from the 15 intervention districts with the following eligibility criteria: female sex, age 18 years or above, history of having sex (vaginal, anal, or oral) at least once within the last month with reward (gifts, money, items, etc), duration of at least 6 months since the last HIV test, not currently planning or receiving HIV-related care at a health service, negative HIV report or unknown HIV status, not currently participating in another HIV-related study, and agreement to participate in the study.

FSWs in the control arm were reached through routine community-based outreach and health promotion package as those in the intervention districts, with the exception of OFT, which was only available in the intervention districts.

Due the web-based component of this study, participants are those who have access to the internet either through their own mobile devices or computers, or through OWs’ devices.

### Study Kits and Media

In order to better promote the intervention on digital platforms, the name *Tes Mandiri Komunitas*, which means community self-testing, was abbreviated to *Teman Kita*, which means “our friend.” A logo bearing the name (Multimedia Appendix 1) was attached to digital and printed material used for the intervention arm.

**Figure 3:**
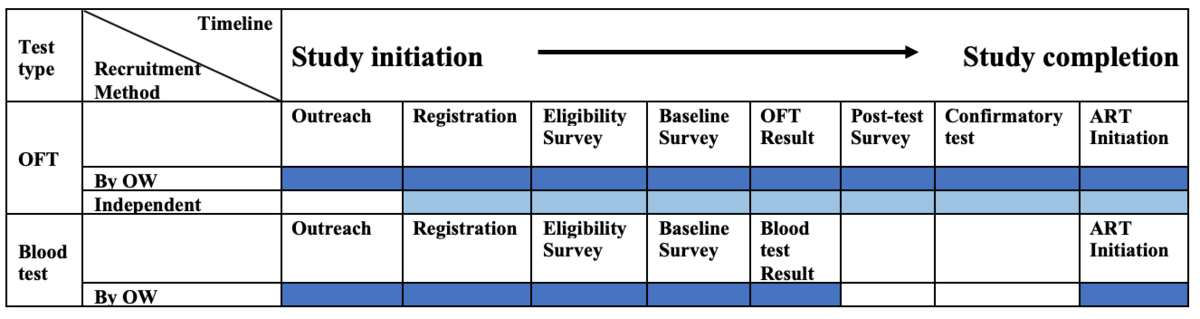
Timeline of the intervention arm showing processes for different types of HIV tests chosen. ART: antiretroviral therapy; OFT: oral fluid test; OW: outreach worker.

This trial used OraQuick ADVANCE Rapid HIV1/2 Antibody Test kits to screen oral fluid samples, which has been shown to have high sensitivity and specificity [39,40]. The study kits included an OFT kit; an instruction booklet in Indonesian adapted from the product insert; another booklet on further instructions for positive test results; a card containing the contact information of the principal investigator, study coordinator, and office; a referral form for confirmatory testing at a health facility; and an ART initiation form (Multimedia Appendix 2). All study kits were labelled with an ID indicating the district and kit serial number on the OFT packaging and on the external packaging (Multimedia Appendix 3). The study process for each participant can be seen in Figure 3. Participants were able to complete the study process at their convenience. Those recruited by OWs were followed up as desired by OWs, and those enrolled independently were followed up by researchers until they completed all steps or until they ceased responding.

Two videos were created to facilitate participants’ and OWs’ use of the study kits. One video provided information for the informed consent, outlining the purpose, process, and reward of the study. This video was only made available to those who were deemed eligible on the website’s eligibility checklist (see Data Collection). The other video was a tutorial outlining the contents of the study kits, instructions for the OFT kits, and instructions for using the web-based data collection system (Multimedia Appendix 4). Institutional affiliations were indicated on the informed consent form and on the study website.

### Sample Size Calculation

In order to detect a 10 percentage point difference in the rate of HIV testing between FSWs choosing assisted and unassisted OFT methods in intervention districts and have (1) 95% certainty that a difference of this magnitude would not have occurred by chance and (2) 90% certainty of detecting a difference of this magnitude if the difference was “real,” the sample of FSWs needed for the intervention districts was calculated using the formula in Multimedia Appendix 5 [41].

The required sample size was n ≥ 635 per OFT method and was increased to n ≥ 761 to allow for 20% loss to follow up. To assess whether “assisted” or “unassisted” OFT results in a larger increase in the rate of facility-based HIV testing, a sample size of n ≥ 761 receiving an assisted or unassisted OFT was necessary; thus, a sample size of n ≥ 1522 participants in the intervention districts was required. All the FSWs reached during the study period using standard of care in the intervention and control districts will be accounted for the analysis.

Sample size requirements also considered the number of participants who tested positive through facility-based testing and were therefore eligible to initiate treatment. This depended on (1) the number of FSWs presenting at health facilities for testing and (2) the positivity rate among those tested. Based on an assumption of a 3% positivity rate, the expected number of participants eligible to initiate treatment will be small and lack sufficient statistical power to make meaningful comparisons with the control arm. For this reason, the impact of the OFT was measured using data from *Sistem Informasi HIV AIDS* (SIHA) in intervention and control districts, which is a database for reporting HIV and AIDS cases in Indonesia.

### Recruitment

#### Outreach Workers

OWs in 15 districts were trained by research staff on the study protocol and use of the web-based data collection system. Trainings were conducted between April 15, 2020, and May 19, 2020. Trainings were initially designed to be conducted in person, but due to the COVID-19 pandemic, trainings were carried out virtually through Zoom for 14 districts and in-person for one district, as the organization conducting the study is also located in the same city. Prior to training, each OW and implementing unit coordinator was given a manual for the website, a practice OFT kit for each implementing unit, spare print material for the study kits, and packaged study kits. Two additional tutorial videos were developed to assist the implementing unit coordinators and OWs in operating the data collection system. These videos were distributed through WhatsApp to each district’s chat group so that individuals could easily store the video in their phones. The link to the developer version of the website was also given prior to training so that implementing unit staff could practice beforehand. Each virtual training session lasted two working days. Interactions with supervision of OWs and implementing unit coordinators’ use of the website were made possible using the screenshare feature on Zoom. Each research staff was responsible to oversee three implementation districts along with one to two control districts, and conduct additional communications and meetings periodically as needed.

#### Social Media Campaign

*Teman Kita* utilized social media platforms to engage with its audience and reach FSWs who were unreached by conventional outreach methods. This approach arose from the understanding that closures of *lokalisasi* in recent years have prompted FSWs to shift their service advertisements online [42]. The COVID-19 pandemic further drove FSWs to seek clients online, as establishments, such as karaoke bars and spas, were forced to operate for limited hours or close down completely [43]. Furthermore, social media has previously been used as a method of recruiting participants from key populations in Indonesia for survey-based studies [44]. Given the covert nature of sex work even on digital platforms [45], it was essential to employ a method that allows FSWs to access study materials and services privately.

A refined digital marketing job posting was formulated in June 2020, and a digital marketing team was recruited to create content, perform analysis on content performance, and generate targeted advertisements. Paid promotions were created to increase awareness toward HIV, testing, and the study, and divert traffic to the study website. Similar strategies of participant recruitment have also been utilized among key populations [46,47], resulting in improved engagement. In addition to creating routine image or text posts, interviews with HIV-positive FSWs and professionals, such as doctors and sexologists, and testimonies on the OFT were obtained and then adapted into posts. OWs were also encouraged to incorporate social media content into their virtual outreach efforts through personal social media accounts or forward information to known FSW networks through WhatsApp messaging chain and dating applications commonly used for sex work. All material published on social media can still be accessed on Facebook, Instagram, and Twitter through the media handle @studitemankita.

#### Satelit Pengambilan Kit OFT/Satellite OFT Pickup Point

The satellite OFT pickup point (SPOT) was a location identified by implementing unit coordinators in the corresponding districts as venues frequented by local FSWs. Although conceived early in the protocol development, this enrolment strategy was only implemented halfway during the data collection in an attempt to recruit more FSWs to access and perform the OFT independently. One SPOT was established in East Jakarta, overseen by a person in charge who stored the study kits and was responsible to refer FSWs to the study website.

### Data Collection

There were two sources of data for this study. The first was the web-based data collection system (Multimedia Appendix 6 and Multimedia Appendix 7), and the second was the outreach data from each implementing unit. The use of the web-based data collection system was informed by the concept of telehealth, which has previously been used to combine home-based testing and video-based consultation to enhance linkage to care among MSM [34] and gender-diverse youth [35]. This approach was able to overcome barriers to facility-based services, suitable for those who fear stigma and live far from services. For our purposes, an external web developer was recruited to develop a web-based data collection system with consultation from key stakeholders. Prior to the development of this system, a survey was conducted among 15 intervention districts to assess regional telecommunication quality and availability, as well as the capabilities of OWs in operating smartphones. Initial plans to develop a smartphone app similar to one described in the study by Biello et al [48], which would allow offline data input, was eliminated due to time constraints.

All instruments used in the intervention of this study were placed on the web-based system, which included the informed consent, baseline survey, posttest survey, and features to upload images of OFT and blood or confirmatory test results (Multimedia Appendix 8). Participants could provide consent digitally by selecting the “agree” button on the informed consent page after correctly answering a series of questions to indicate their understanding of the study. Districts experiencing difficulties in operating the web-based system due to telecommunication limitations were given digital copies of the surveys so that the data collected could be entered in the web-based system when conditions were favorable. A developer version of the website was created to facilitate refining through consulting with stakeholders to ensure the official version was user friendly. One type of participant account and three types of administration user accounts (researcher, OW, and implementing unit coordinator) were created. Participants were able to terminate their participation or withdraw from the study at any time, and these options were reflected in each webpage in the participant’s account.

The second data source involved routine outreach at implementing units, which included names (or pseudonyms) of FSWs, age, and information regarding the HIV care cascade (whether they were offered HIV testing, whether they agreed to test, whether they received testing, and whether ART was initiated for those who tested positive). In order to measure the proportional increase in FSWs who received HIV testing through the OFT in the intervention districts, data from both the website and implementing unit outreach will be combined. These data were collected as a quantitative measure to compare HIV care cascades between intervention and control districts.

### Data Management

The intervention that included the web-based system was available to participants at no cost. The system was password protected, ensuring only those who were authorized had access, and participant privacy was maintained. Participants were also given user names to maintain their confidentiality. Only the investigators were able to access the full information, including surveys of each participant. OWs were only able to view the contact details and progress of the participants they reached, and implementing units were only able to view the contact details and progress of the participants in their districts. The funding agency was not given access to participant information.

The web-based data could also be downloaded as Excel spreadsheets only by investigators. A daily cascade was used to monitor data collection progress and aid analysis (Multimedia Appendix 9). Research staff verified all incoming data to ensure all the necessary information was obtained. They also clarified conflicting information with OWs and performed random checks with participants through WhatsApp messaging or phone calls to ensure the intervention was performed according to the procedure. Participants were also verified against implementing unit outreach data to ensure that there was no documentation of accessing HIV blood testing services within the past 6 months. A second check against implementing unit outreach data was also performed by the data manager after participants were verified by the research staff. After all data were verified, participants were given their incentives.

### Data Monitoring

An independent data monitoring committee was not established as the trial aims to understand the acceptance of a test kit that is already widely used in other parts of the world. Progress and challenges in data collection were communicated and resolved between the research team and stakeholders continuously. The recruited sample size was constantly monitored to evaluate kit redistributions, recruitment decisions, and termination of data collection. Decisions were made based on consultations with the principal investigators, funding agency, and implementing partners. Researchers also monitored the progress of each participant and notified OWs to follow up with participants who had not completed their intervention process and to directly communicate with participants who enrolled through social media.

Undesired events related to the testing process were communicated among OWs, research staff, and principal investigators. These events included participants who encountered difficulties in proceeding with the testing process, duplicate enrolments, and participants found to be ineligible. Decisions regarding follow-up actions were consulted and made with the principal investigators and implementing partners.

### Outcome Measures and Statistical Methods

#### Primary Outcomes

To measure the differences between control and intervention districts, two proportion *t* tests will be performed to compare the primary outcomes of this study, which are HIV testing uptake, HIV positivity rate, and ARV initiation rate. Specific daily quantitative data collected to answer the primary outcomes can be found in Multimedia Appendix 9, which outlines the different information that could be gathered from participants in control and intervention districts.

#### Secondary Outcomes

The secondary outcomes examine characteristics and behaviors among FSWs accepting the assisted and unassisted OFT and the perception of these approaches. Questions on demographic characteristics, condom use, client base, history of sexually transmissible infections, and Bahasa Indonesia version of the 12-item short HIV stigma scale [49] were present in the baseline survey. Additionally, perception of the experience of OFT use was present in the posttest survey (Multimedia Appendix 10). Chi-square analyses will be used to evaluate the secondary outcome measures.

**Figure 4:**
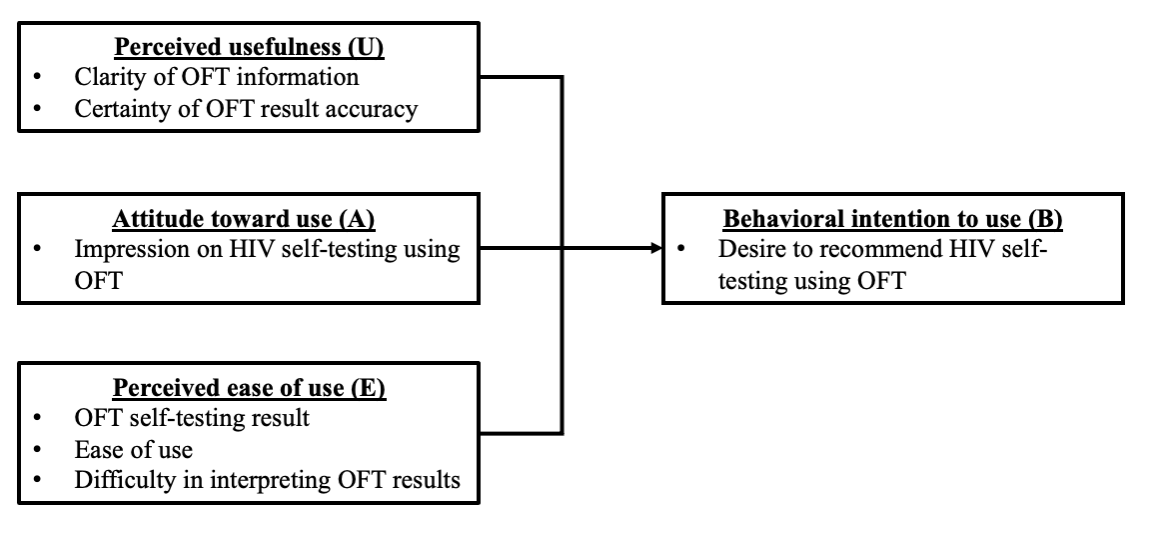
Adapted Technology Acceptance Model framework for analysis of the perception toward oral fluid test (OFT) use.

Analysis on the perception of OFT use will be performed using an adaptation of the Technology Acceptance Model (TAM) Framework [50]. Questions on the posttest survey were separated into TAM categories of perceived usefulness, attitude toward use, and perceived ease of use to determine the behavioral intention of using the OFT in the future (Figure 4). Each question can be answered using a Likert scale with a score range from 1 to 5 (1 indicating extreme disagreement with the statement and 5 indicating extreme agreement with the statement).

### Ethics Approval

The study protocol was reviewed and approved by the Faculty of Medicine, Udayana University-Sanglah General Hospital Ethics Committee with the number 612/UN14.2.2.VII.14/LT/2020. Research permission was granted by the Department of Internal Affairs, Republic of Indonesia with permit number 440.02/145/DV, which was subsequently referred to the licensing office in each province and priority district. This study has been registered with ClinicalTrials.gov (NCT04578145). No amendments to ethics were made during the trial.

During the trial, a need for qualitative process evaluation was identified by key stakeholders. The qualitative study was performed separately by separate researchers under a different ethics approval. Procedures within the qualitative study will not be discussed under this protocol.

## Results

Commencement of CBS coincided with the emergence of the COVID-19 pandemic, which prompted changes to alternate modes of OW training, outreach methods, data verification, and participant recruitment, fully utilizing virtual communication technologies. Initiation of data collection was staggered through 15 intervention districts between April 20, 2020, and May 29, 2020, to accommodate different timings in OW virtual training. Data collection in control districts commenced simultaneously in April 2020. All data collections ended on October 31, 2020.

The shift to a virtual modality in training OWs and monitoring of study implementation was conducted with great success. All OWs involved in the study attended the virtual trainings prior to the study initiation and were given guidance by research staff on the web-based system and relevant study procedures throughout the data collection period. The outreach effort using social media was also able to facilitate participant recruitment such that the targeted sample size in the intervention district was fulfilled. Furthermore, promoting the intervention through social media allowed the information to reach a wider audience in addition to utilizing the network of OWs.

In the intervention districts, 1907 FSWs completed the informed consent and 1545 opted to undergo the OFT (1516 assisted and 29 unassisted), with an overwhelming number (n=1518) enrolled through OWs, 27 enrolled independently through social media, and none enrolled through SPOT. There were 428 people who attempted to register independently through social media, but not all identified as FSWs; hence, many were ineligible for the trial. A number of these participants registered using names that are typically assigned to males in Indonesia; however, because the questionnaire did not have the option of choosing sex, it was not possible to definitively conclude that these participants were male. Furthermore, there were registrants who reached out to the research team after they were deemed ineligible by the questionnaire. Some reported being sexually active with their partners but did not participate in sex work.

The use of SPOT and virtual outreach by OWs were attempted to harness the strengths of conventional outreach that have been successful in the past. As such, this method of recruitment was not promoted on the study’s official social media channels. Surprisingly, however, no participant was recruited through SPOT despite it being a location identified to be frequented by local FSWs.

In the control districts, data were routinely submitted at the end of each month. The completeness of the data was verified by research staff with the assistance of implementing unit coordinators. As control districts maintained their practices in the HIV program, only quantitative data were relevant for the purposes of answering the primary objectives in this trial.

Three forms of research dissemination are planned as follows: (1) a detailed report for the study sponsor, (2) presentations for stakeholders, and (3) a research article to be submitted to an open-access peer-reviewed journal. A brief version of the study protocol was uploaded to the implementing organization’s website. The data generated will be disseminated to each implementing unit.

## Discussion

### Preliminary Insights

The trial was able to recruit the required sample size. Preliminary results indicated that uptake of HIV testing at health facilities among FSWs in the intervention districts was slightly higher compared to the control districts. The HIVST strategy using the OFT was well received and was able to recruit participants through social media. This indicates the feasibility and acceptability of a web-based HIVST strategy to increase testing uptake among FSWs in Indonesia.

This study demonstrated cutting-edge implementation research to assess possible scaling up of HIVST using the OFT at the national level. This study is performed at a larger scale than former studies on FSWs in Indonesia, and the processes of training, implementation, and monitoring were performed almost entirely online. This indicates that telecommunication infrastructure in urban areas is adequately developed and the use of smartphones is widespread, and points to the readiness for implementing telehealth at a larger scale. The wide reach and utilization of courier services, whether through regular postal services or rideshare companies with delivery services, are recognized. This opens the possibility for fully online modalities of test and treat services, which has never been previously attempted in Indonesia. Learning from recent key population-led initiatives in Bangkok, Thailand, adaptations were made to service delivery in light of COVID-19 [51]. These included the introduction of telehealth, self-sampling and testing for HIV and sexually transmissible infections, and pre-exposure prophylaxis medication delivery using courier services [51].

The study design was also appropriate because it allowed FSWs to perform assisted or unassisted testing and test at their convenience, as many clinics and community health centers were operating at limited capacity in addition to social restrictions at some of the intervention districts. The social media campaigns and web-based data collection methods were innovative approaches to supplement conventional outreach. As FSWs in Indonesia shift toward online methods to obtain clients [5], utilizing social media and web-based data collection can potentially capture previously unreached or hard-to-reach FSWs. Similar study designs that utilize internet-based interventions for self-testing were used mostly among MSM [34,36,52,53] and indicated the promising potential of using this approach to increase HIV uptake [52-54]. However, similar study designs for FSWs are yet to be implemented, which makes this study crucial in understanding the feasibility and acceptance of similar approaches in increasing HIV uptake among FSWs.

### Limitations

Despite the multifaceted approach in recruiting FSWs, an overwhelming number of participants were recruited by OWs as opposed to registering independently through social media, which was likely due to the relatively late preparation and initiation of professional digital marketing efforts. However, this is consistent with findings from an HIVST trial among men and adolescents, where the majority of participants (82.7%) were recruited through community-based distributors [55], pointing to the crucial role of OWs in outreach. Additionally, the unknown digital footprint of FSWs posed a great challenge for creating targeted advertisements and social media campaigns. However, there was a notable increase in independent enrolment, although not all were eligible for the trial. Although the involvement of OWs was not systematically embedded in the study’s social media campaign, it is important to note that OWs routinely utilized other social media platforms to engage with FSWs. Similarly, another HIVST trial among key populations, including FSWs, also found that 77.6% of participants opted for lay-provider testing instead of self-testing [25], which translates roughly to assisted and unassisted methods in our study. Given the covert nature of sex work, even online [45], further digital marketing research and strategies involving OWs must be incorporated to tailor messaging and engagement with FSWs in Indonesia.

The recruitment process through SPOT was unsuccessful, possibly due to business and mobility restrictions in Jakarta, which limited activities at nonessential establishments. Promotion of HIVST through SPOT relied solely on the person-in-charge’s personal networks and may have narrowed the possible reach of the information. Since SPOT was not trialed in other districts with fewer restrictions, it was not possible to systematically assess the effectiveness of this method in this trial.

An additional limitation was the eligibility criteria applied in this study, which required participants to have exchanged sex for reward within the past month. This parameter may have ruled out FSWs who have had challenges obtaining customers during the COVID-19 pandemic. During the pandemic, many people lost their jobs and had to return to their hometowns [56]. Likewise, as the intervention measures were implemented in certain priority districts, FSWs who left these areas to return to their hometowns were unable to receive the intervention provided through this study.

Furthermore, as our study maintained a “business as usual” approach to the programs in control districts, the data collected were limited. For intervention districts, only those who were eligible filled out surveys, while other FSWs also received standard of care. Therefore, data are limited because demographic information, risk behavior information, and perception of stigma toward HIV/AIDS were only collected from those who fulfilled the eligibility criteria. As such, we will not be able to make comparisons between the characteristics of FSWs in the intervention and control districts.

### Further Research Needs

The combination of innovative approaches offered a timely response to Indonesia’s evolving sex work industry in an increasingly digitized nation. This study is the first to employ a combination of outreach and recruitment methods, as well as data collection methods, to ensure service delivery for HIV testing stays relevant and accessible in the digital era. The CBS protocol provided a holistic approach in reaching FSWs across Indonesia. Furthermore, CBS also increased the implementation sites to 15 intervention and eight control districts, providing insights that represent different urban settings across the archipelago, compared to a previous OFT study among key populations, which was implemented in three major urban centers in Java [28]. Given that there were no documented social media campaigns targeting FSWs for study recruitment in Indonesia, CBS successfully pioneered a large-scale targeted virtual outreach in Indonesia for this population that potentially lays the foundation for the future of HIV prevention outreach among FSWs.

Just as interesting is the finding that there were people who did not identify as FSWs but registered independently through the study’s social media campaign. This suggests that the OFT is not only a tool that can increase testing among FSWs, but also a tool that captures the interest of those who perceive they are at risk for HIV due to sexual practices. The reach of social media also goes on to demonstrate the possibility to reach FSW and non-FSW populations who are not reached through conventional HIV program outreach.

### Conclusion

This trial indicated that utilizing virtual methods in training OWs, utilizing outreach on social media, and adding the OFT to the standard of care in HIV programs are feasible. It also highlights the important role of OWs in facilitating access to online services and in using OFT kits. Likewise, it is important to understand how to better engage and reach the target population through social media. However, since most participants were recruited through OWs and opted for an assisted OFT, it will be challenging to understand differences between FSWs who were recruited through different methods and who opted for different testing methods. Regardless, the large number of participants opting for the OFT and the success in attaining the required sample size through combined traditional and virtual outreach point to the possibility of promoting routine testing with this modality.

The multidimensional and interdisciplinary approach aims to optimize the effectiveness of HIVST in increasing testing uptake and seeks to provide information on the profile of participants who access the OFT and preferred methods of testing. The administration of posttest surveys also acts as user feedback regarding satisfaction on specific aspects of OFT delivery and its potential to increase testing and linkage to care. The results from this study as a randomized community trial will provide robust and valuable evidence that can be used in advocating and planning for significant scaling up of HIVST among FSWs in Indonesia over the next few years. 

## Appendix

Multimedia Appendix 1 Logo of the trial used for branding in the intervention arm.

Multimedia Appendix 2 Screenshot in the instructional video for the oral fluid test showing the contents of the study kit.

Multimedia Appendix 3 A study kit with the study logo and kit ID bearing the district code and kit number.

Multimedia Appendix 4 Instructional video on the use of the website and oral fluid test for study participants.

Multimedia Appendix 5 Formula for calculating sample size.

Multimedia Appendix 6 Screenshot of the study website's homepage with informative articles on HIV, women's health, and sexual reproductive health.

Multimedia Appendix 7 Screenshot of the information page on the study's website with an instructional video, clinic locations, and relevant study information.

Multimedia Appendix 8 Text of questionnaires on the study website.

Multimedia Appendix 9 Quantitative data monitored in the daily cascade.

Multimedia Appendix 10 Data collected for the primary and secondary outcomes.

## Abbreviations

ART: antiretroviral therapy
CBS: community-based HIV screening
FSW: female sex worker
HIVST: HIV self-testing
MSM: men who have sex with men
OFT: oral fluid test
OPSI: Organisasi Perubahan Sosial Indonesia
OW: outreach worker
SPOT: Satelit Pengambilan Kit OFT (Satellite OFT Pickup Point)
TAM: Technology Acceptance Model
WHO: World Health Organization
