# Supplementary figures and images for: Web-Based Multifaceted Approach for Community-Based HIV Self-Testing Among Female Sex Workers in Indonesia: Protocol for a Randomized Community Trial
Source: JMIR Res Protoc. 2021 Jul 21;10(7):e27168. doi: 10.2196/27168 (PMC8339988; doi:10.2196/27168)

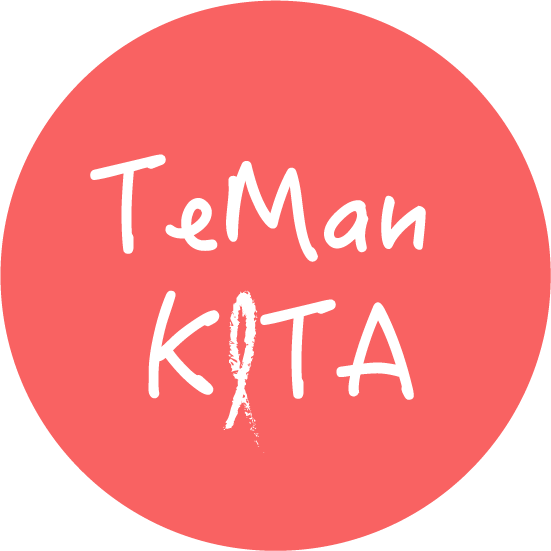

Supplement: Multimedia Appendix 1 [file resprot_v10i7e27168_app1.png]

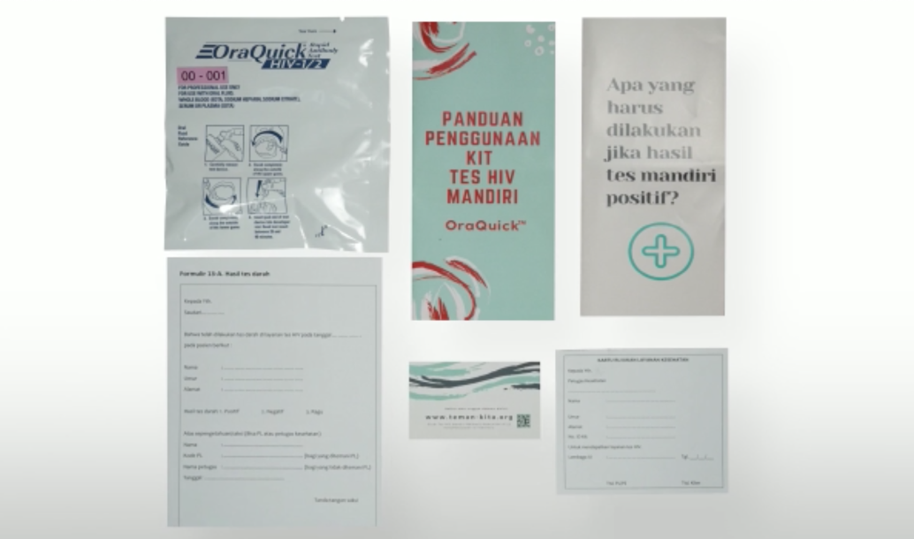

Supplement: Multimedia Appendix 2 [file resprot_v10i7e27168_app2.png]

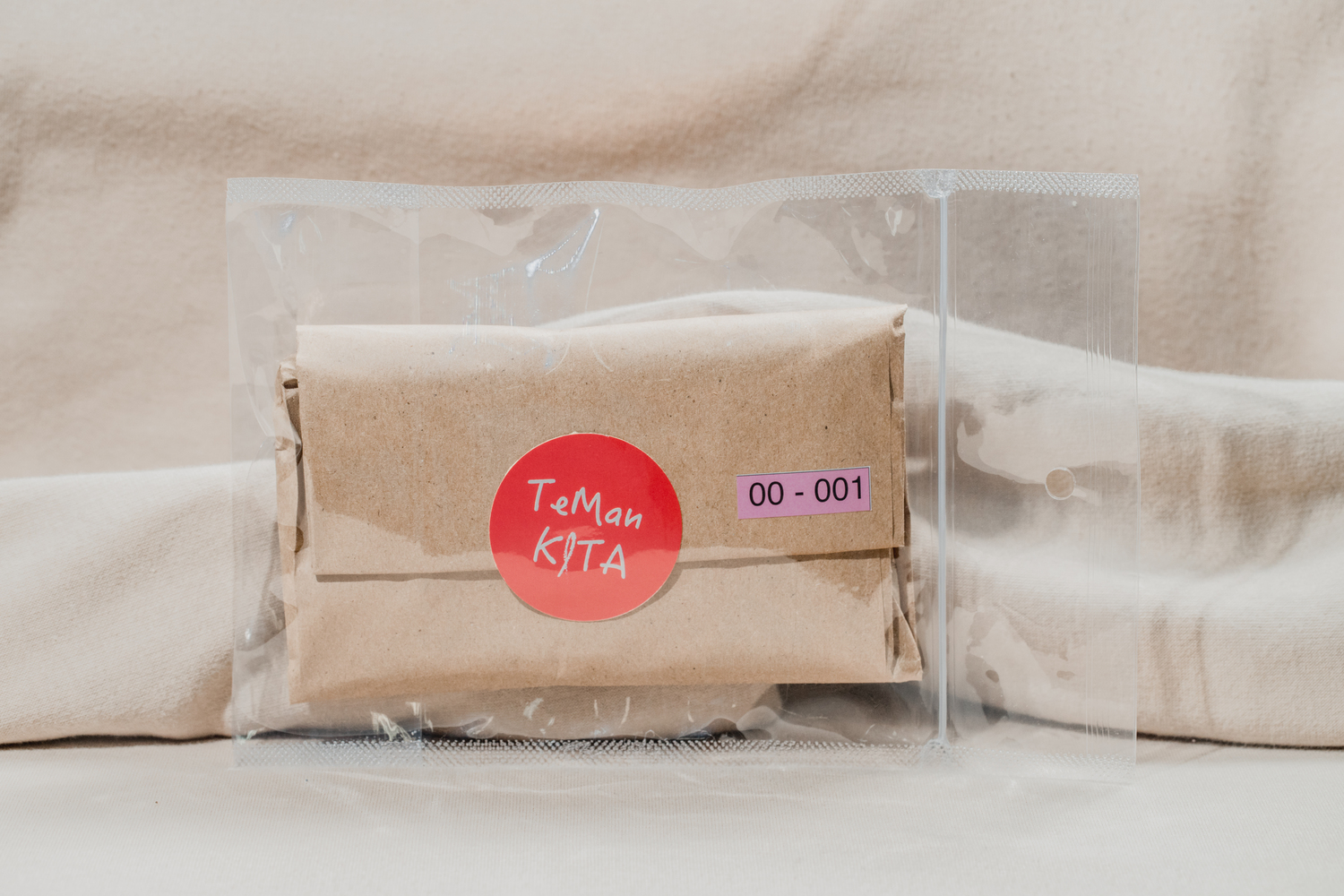

Supplement: Multimedia Appendix 3 [file resprot_v10i7e27168_app3.png]

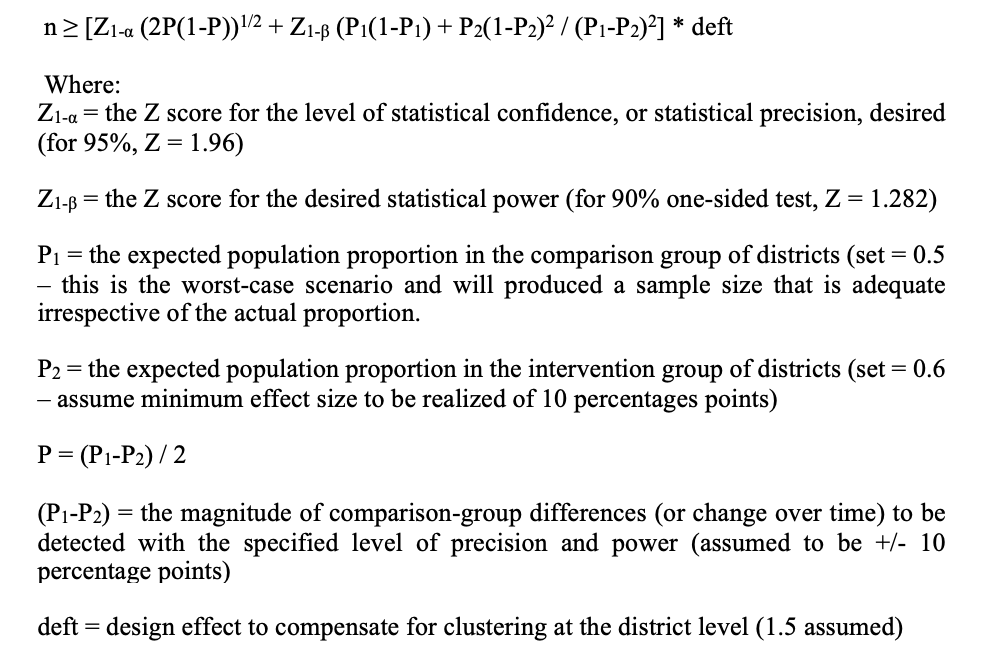

Supplement: Multimedia Appendix 5 [file resprot_v10i7e27168_app5.png]

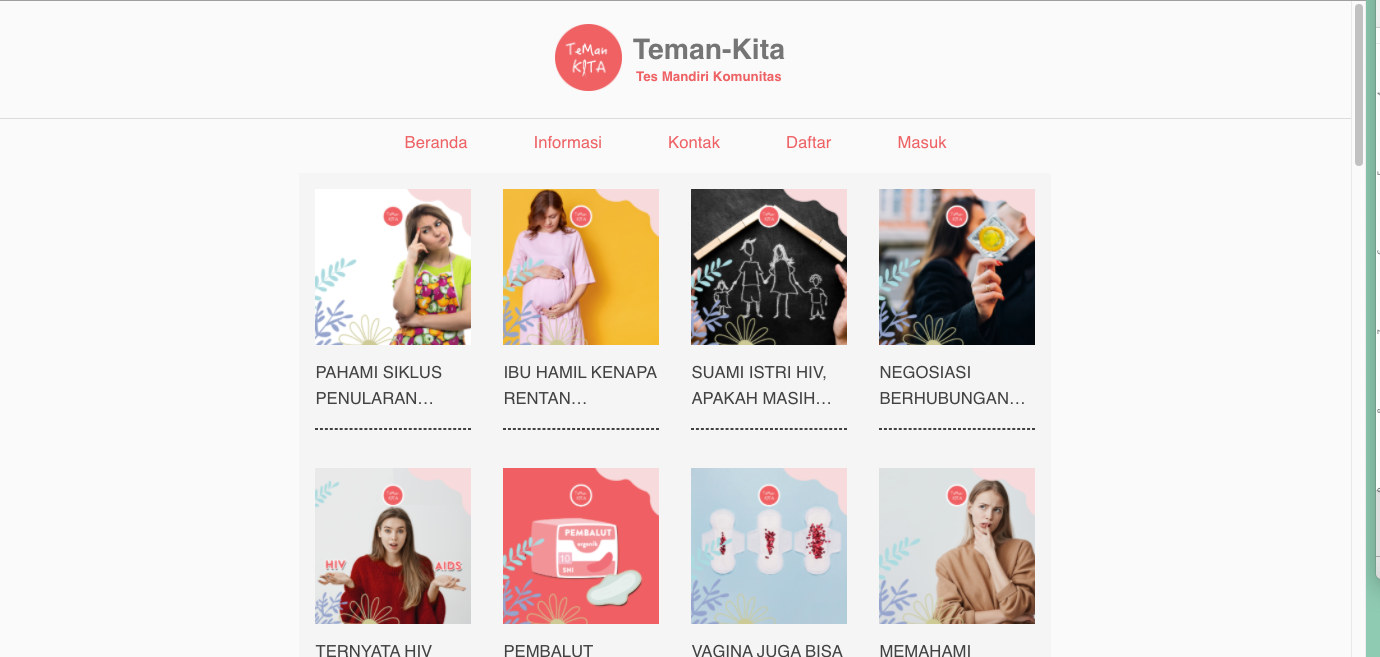

Supplement: Multimedia Appendix 6 [file resprot_v10i7e27168_app6.png]

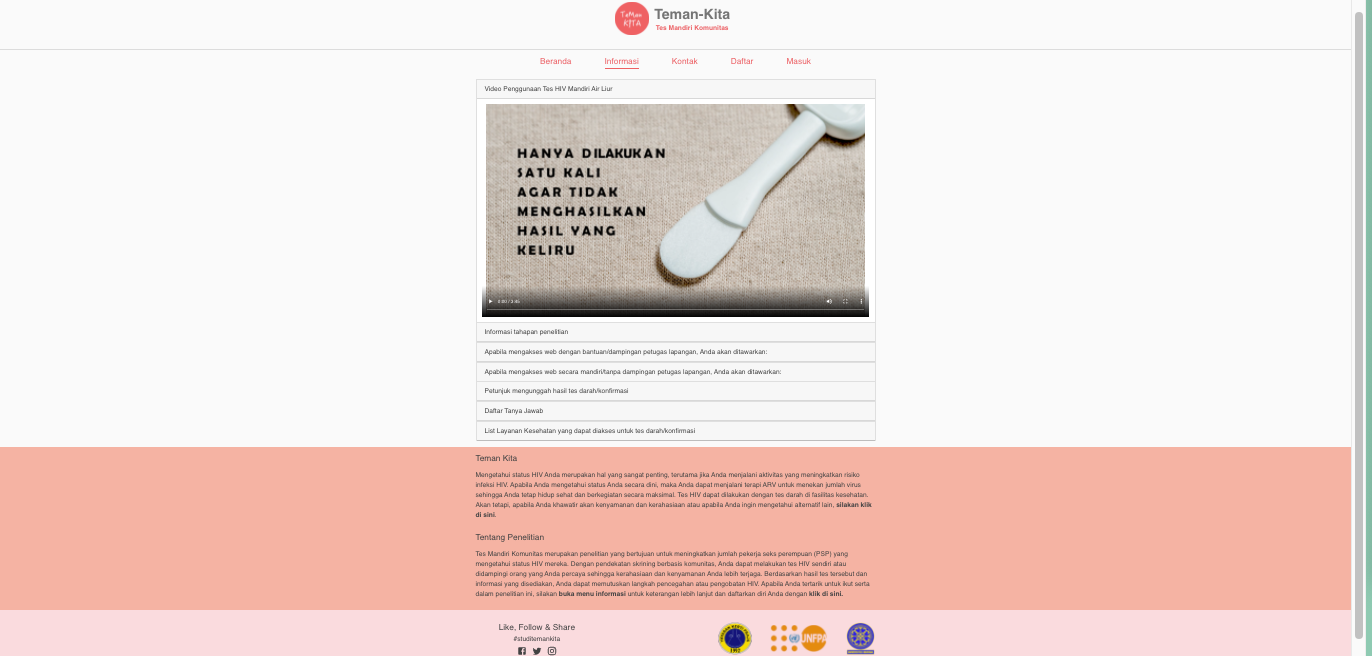

Supplement: Multimedia Appendix 7 [file resprot_v10i7e27168_app7.png]
