# Supplementary material for: Web-Based Multifaceted Approach for Community-Based HIV Self-Testing Among Female Sex Workers in Indonesia: Protocol for a Randomized Community Trial
Source: JMIR Res Protoc. 2021 Jul 21;10(7):e27168. doi: 10.2196/27168 (PMC8339988; doi:10.2196/27168)
Supplement: Multimedia Appendix 8 [file resprot_v10i7e27168_app8.docx]

**Outreach Form**

| **Outreach information** | | |
| --- | --- | --- |
| Full name |  | |
| Place of birth |  | |
| Date of birth |  | |
| Type of FSW | Direct | Indirect |
| Cellphone number |  | |
| Last HIV test | (Months/Years) | |
| Offered HIV test by OW? | Yes | No |
| Agreed to perform HIV blood test? | Yes | No |
| Came for HIV blood test? | Yes | No |
| IU City/Regency |  | |
| Date of outreach |  | |

**Eligibility Form**

| **No** | **Criteria Inclusion** | **Yes** | **No** |
| --- | --- | --- | --- |
| 1 | Female, 18 years old or over |  |  |
| 2 | Recognizing having sex (vaginal, oral, or anal) at least one time during the last month with receiving money or others |  |  |
| 3 | Never be involved in testing HIV during the last 6 months |  |  |
| 4 | Recognizing that the HIV status is negative or positive |  |  |
| 5 | Be ready to participate in this study |  |  |
| 6 | Never being participate in other studies focusing on HIV prevention |  |  |

| 7 | Not visiting a health service for an HIV test or not visiting a mobile test |  |  |
| --- | --- | --- | --- |
| 8 | Have been regularly undergoing HIV blood tests |  |  |

**Study Information (Text of informed consent video)**

Hello, best wishes for all of us. I will explain to you about a research conducted in 23 districts in Indonesia, which aims to encourage more friends who work like you, to carry out HIV testing with HIV screening which is done by yourself.

You are invited to participate in this research, because you meet the criteria to be participant as follows:

- Aged 18 years or older
- Has worked as it is now for at least the past one month
- Have not taken an HIV test in the past six months or have never had an HIV test
- You don’t know your HIV status

If you agree, you will be asked to provide a sign of agreement on the form provided. Your participation in this research is voluntary and you can cancel your participation whenever you want.

In this study you will be asked to fill in or answer a number of questions before and after the test.

For the type of test, you may choose a blood or saliva test. If you choose a blood test, you are asked to come to the health facility to do the test. Next you are asked to submit the test result to us. If you choose the saliva test, you can choose the test with the assistance of an officer or the test that is done independently. After doing the saliva test, you are asked to report the result of the test and answer a few questions.

If your saliva test results are positive or doubtful, you are asked to do a confirmation test at the health facility. If the test results are positive, you are advised to take ARV treatment immediately. If the results of the saliva test are negative or unclear or doubtful, you can still do the confirmation test at the health facility.

By joining this research you will know your HIV status and you will continue to live a healthy and productive life. In addition, you have contributed in increasing knowledge and assisting the Government of Indonesia to improve HIV and AIDS prevention and control programs.

We provide incentives to cover your transport and time in participating in this study:

• IDR 50,000 each for the initial interview or answering questions before and after the test

• IDR 100,000 for undergoing a blood test and reporting the result.

• IDR100,000 for doing a saliva test and reporting the test result.

• IDR 100,000 for performing a confirmation test and reporting the result.

Your participation and personal data will be kept confidential. Only people who are related to the research who know about your participation. They are researchers, field officers, and medical officers. They will not publish your personal data without your consent.

When you participate in this research, you need to spend time answering questions or conducting tests. You may feel uncomfortable when answering questions or when knowing the results of the test. There may also be a risk of injury in taking blood, but health workers are trained and experienced so that the risk is very small

If you need further information, you can contact:

1. Principai Investigator: dr. Pande Putu Januraga, M. Kes, DrPH.

(+62 812 4618 0389)

1. Research coordinator: I Gusti Agung Agus Mahendra, MPH

(+62 857 9236 0767)

1. Health Research Etic Commissions of The Medicine’s Faculty of Udayana Universities/ Sanglah Hospital

(0361) 22791115)

Thank you for paying attention to the information on this video. We look forward to your participation.

**Assessment of participant’s understanding on the research (available on the website)**

| No | Statement | Answer | |
| --- | --- | --- | --- |
|  |  | Yes | No |
| 1 | I already understood the purpose of this research and why I was asked to participate. |  |  |
| 2 | I have understood that participation in this research is voluntary |  |  |
| 3 | I already know what I was asked to do for this research, if I agree to participate |  |  |
| 4 | I already understand that I get a number of incentives if I agree to participate |  |  |
| 5 | I have understood that my participation and personal data will be kept confidential by the research team |  |  |
| 6 | Before deciding to take part in this research, I was given enough time to consider and ask if there was anything I did not understand |  |  |
| 7 | I understand that if I have any questions about this research I can contact the contacts provided. |  |  |

*If not fully understood, please read again the information above or contact our call center*

*0821-4616-5389 (phone/WhatsApp)*

**The Agreement to Participate to The Study**

**The research of community-based screening HIV self-testing for the female sex workers in 23 prioritized districts or cities in Indonesia**

1. I am .................................................................................... living in………………………………………………………

agree to participate as the participant of the study that is explained on the form information participation is explained above (or it is attached on this form)

1. I acknowledge that I have read the form participants information, who explained why I have been chosen, the aim of the study, the nature and the possible risk of study, and the information form has given the explanation in detail, so I do understand it clearly.
2. Before signing this agreement, I have been given a chance to ask some questions in terms of each mental and physical risk which is possible I suffers as the consequence of participating and I have received the satisfied answers.
3. I understand that I can withdraw from this study anytime without influencing my relationship with health services of Non-Government Organization (NGO) who accompanied me right now.
4. I agree that the research data which is collected of this study can be published with give protection of my identity.
5. I agree that If I have questions concern on taking part as participants of this study, I can inform dr. Pande Putu Januraga, M. Kes, DrPH in +62 812-4618-0389, which is glad to answer my questions.
6. I acknowledge to receive a copy of the form agreement and the form of participant information.

Complaint can be informed to Yayasan Kerti Praja, Phone: (0361) 728916.

| I agree | I disagree |
| --- | --- |

**Introduction**

Thank you for your willingness to participate in this study and to answer this simple questionnaire. Your participation is greatly valued. Allow us to provide some more information regarding the survey.

Firstly, this study is part of a national study in evaluating the most appropriate method for HIV testing for female sex workers who are at least 18 years of age, have never been tested for HIV and have not undergone HIV testing in the last 6 months but would like to receive HIV blood testing at this time.

Secondly, your participation and personal information will be kept **confidential**. Only the research team will know you, and you will not be writing your name or any identifying information explicitly on this questionnaire.

Additionally, only the research team will know your answer. Each questionnaire has been given a code, such that your personal details will be safe and can only be accessed by the research team.

Thirdly, the findings in this study will be presented as a collective result, such that personal information will not be presented, including yours.

Fourthly, you may refuse to answer the questions given, however we will greatly appreciate if you answer all the questions, as it will assist us in generating better results.

Should you have doubts regarding a particular question or certain words in this questionnaire, do not hesistate the **contact us** **(at OW/PE’s phone number: ________________________)** for further explanation. There is no right or wrong answer, please provide that answer that you consider most accurate.

Are you ready to begin?

| Yes, I am ready! |
| --- |

**Baseline Survey for All Participants (Blood testing at healthcare facility and HIV self-testing)**

| Participant Code Number | |  |
| --- | --- | --- |
| Name of OW/PE (assisted) | |  |
| 1. | Please write your full name or initials |  |
| 2. | What is your date of birth? | Day: ___Month: __ Year : ____ |
| 3. | Your age is | ……years old |
| 4. | What was the education level you last received? | 1. Did not attend school |
|  |  | 1. Did not complete elementary school/ equivalent |
|  |  | 1. Completed elementary school/equivalent |
|  |  | 1. Did not complete middle school/equivalent |
|  |  | 1. Completed middle school/equivalent |
|  |  | 1. Did not complete high school/equivalent |
|  |  | 1. Completed high school/equivalent |
|  |  | 1. Ever attended/completed bachelor studies |
| 5. | What is your current marital status? | 1. Never married |
|  |  | 1. Married (including unofficial marriages) |
|  |  | 1. Separated |
|  |  | 1. Widowed |
|  |  | 1. Divorced |
| 6. | Do you have a consistent partner (boyfriend/husband) in the past month? | 1. Yes |
|  |  | 1. No |
|  |  |  |
| 7. | What was your age when you first engaged in sex with a client? | ………….. (years of age) |
| 8. | What is the approximate number of clients you had in the past 7 days? | ………….. (people) |
| 9. | How often do you use condom when engaging in sex with your clients in the past month? | 1. Always |
|  |  | 1. Very frequent |
|  |  | 1. Often |
|  |  | 1. Sometimes |
|  |  | 1. Never |

**Questions on Stigma**

| **No** | **Question** | **Extremely Disagree** | **Disagree** | **Agree** | **Extremely Agree** |
| --- | --- | --- | --- | --- | --- |
|  | **Personalized stigma** |  |  |  |  |
| 1 | Others will avoid touching me if I am infected with HIV |  |  |  |  |
| 2 | I am worried that my family/friends/partner/husband/boss will end their relationship/contact with me if I am infected with HIV |  |  |  |  |
| 3 | I am worried that I will lose my friends if I am infected with HIV |  |  |  |  |
|  | **Concerns with Revealing Status** |  |  |  |  |
| 4 | Revealing to others that I am infected with HIV will pose a risk to me |  |  |  |  |
| 5 | I have to conceal my status if I am infected with HIV |  |  |  |  |
| 6 | I might have to be extremely careful to whom I reveal that I am infected with HIV |  |  |  |  |
|  | **Concerns regarding public attitude** |  |  |  |  |
| 7 | I am afraid that I will be treated like an unwanted person if I am infected with HIV |  |  |  |  |
| 8 | Most people believe that those infected with HIV are dirty |  |  |  |  |
| 9 | Most people feel uncomfortable being around people living with HIV |  |  |  |  |
|  | **Negative Self Perception** |  |  |  |  |
| 10 | I feel at fault if I am infected with HIV |  |  |  |  |
| 11 | Others attitude towards HIV will worsen my feelings if I am infected with HIV |  |  |  |  |
| 12 | I feel that I am not as good as others if I live with HIV |  |  |  |  |

**Post test questionnaire**

| KIT Code | | 0 | 1 | - | 0 | 0 | | 1 | |  |
| --- | --- | --- | --- | --- | --- | --- | --- | --- | --- | --- |
| Participant Code (First 4 letters, year, month, day) | |  | | | | | | | | |
| Name of OW/PE | |  | | | | | | | | |
| 1. | Please write your full name or initials |  | | | | | | | | |
| 2. | How was the clarity of the information about HIV test kit you received/obtained? **(Select one answer)** | 1. Extremely unclear | | | | | | | | |
|  |  | 1. Unclear | | | | | | | | |
|  |  | 1. Fairly clear | | | | | | | | |
|  |  | 1. Clear | | | | | | | | |
|  |  | 1. Extremely clear | | | | | | | | |
| 3. | When you performed HIV self-testing, was there any person (peer) who assisted you? | 1. Yes | | | | | | | | |
|  |  | 1. No | | | | | | | | |
| 4. | How easy was performing an HIV test using the self-testing kit given? **(Select one answer)** | 1. Extremely difficult | | | | | | | | |
|  |  | 1. Difficult | | | | | | | | |
|  |  | 1. Fairly difficult | | | | | | | | |
|  |  | 1. Easy | | | | | | | | |
|  |  | 1. Extremely easy | | | | | | | | |
| 5. | What was the reason that **prevented you from testing** using the test kit provided? | 1. Extremely difficult | | | | | | | | |
|  |  | 1. Difficult | | | | | | | | |
|  |  | 1. Fairly difficult | | | | | | | | |
|  |  | 1. Easy | | | | | | | | |
|  |  | 1. Extremely easy | | | | | | | | |
| 6. | **This question only applies for those who underwent the self-testing assisted by OW/PE.** How would you rate the support given by OW/PE /contact people who accompanied/contacted you during HIV testing independently  (**Select an answer**) | 1. Extremely unclear | | | | | | | | |
|  |  | 1. Unclear | | | | | | | | |
|  |  | 1. Fairly clear | | | | | | | | |
|  |  | 1. Clear | | | | | | | | |
|  |  | 1. Extremely clear | | | | | | | | |
| 7. | This question only applies if your test results are negative. How much do you want to take an HIV test regularly after taking this test? | 1. Extremely not in favour | | | | | | |  | |
|  |  | 1. Less in favour | | | | |  | | |  |
|  |  | 3. Fairly in favour | | | | |  | | |  |
|  |  | 4. In favour | | | | |  | | |  |
|  |  | 5. Very in favour | | | | |  | | |  |
| 8. | How was your experience in performing this HIV self-testing (saliva test)? | 1. Extremely unpleasant | | | | | | | | |
|  |  | 1. Unpleasant | | | | | | | | |
|  |  | 1. Fairly pleasant | | | | | | | | |
|  |  | 1. Pleasant | | | | | | | | |
|  |  | 1. Extremely pleasant | | | | | | | | |
| 9. | How sure are you with the accuracy of this test | 1. Very doubt | | | | | | | | |
|  |  | 1. Less sure | | | | | | | | |
|  |  | 1. Fairly sure | | | | | | | | |
|  |  | 1. Sure | | | | | | | | |
|  |  | 1. Very sure | | | | | | | | |
| 10. | How was your test result? | 1. Positive | | | | | | | | |
|  |  | 1. Negative | | | | | | | | |
|  |  | 1. Indeterminate | | | | | | | | |
| 11. | How much do you want to confirm your test results to the healthcare facilities? | 1. Extremely unlikely | | | | | | | | |
|  |  | 1. Fairly unlikely | | | | | | | | |
|  |  | 1. Fairly likely | | | | | | | | |
|  |  | 1. Likely | | | | | | | | |
|  |  | 1. Extremely likely | | | | | | | | |
| 12. | How likely are you to recommend your peers to undergo the screening test that you just performed? | 1. Extremely unpleasant | | | | | | | | |
|  |  | 1. Unpleasant | | | | | | | | |
|  |  | 1. Fairly pleasant | | | | | | | | |
|  |  | 1. Pleasant | | | | | | | | |
|  |  | 1. Extremely pleasant | | | | | | | | |
| 13 | How much do you trust the accuracy of the test result | 1. Extremely unsure | | | | | | | | |
|  |  | 2. Fairly unsure | | | | | | | | |
|  |  | 3. Fairly sure | | | | | | | | |
|  |  | 4. Sure | | | | | | | | |
|  |  | 5. Extremely sure | | | | | | | | |
| 12. | What was your test result? | 1. Positive 🡺 P.13 | | | | | | | | |
|  |  | 1. Negative 🡺 P.14 | | | | | | | | |
| 13. | If you are extremely unlikely or unlikely to visit a healthcare service, what are the reasons? |  | | | | | | | | |
| 14. | What is your opinion regarding this HIV testing method? |  | | | | | | | | |
| 15. | What are your suggestions for HIV self-testing? |  | | | | | | | | |
| 16. | What are your suggestions and comments to improve the procedure of this study? |  | | | | | | | | |
