# Supplementary material for: Web-Based Multifaceted Approach for Community-Based HIV Self-Testing Among Female Sex Workers in Indonesia: Protocol for a Randomized Community Trial
Source: JMIR Res Protoc. 2021 Jul 21;10(7):e27168. doi: 10.2196/27168 (PMC8339988; doi:10.2196/27168)
Supplement: Multimedia Appendix 9 [file resprot_v10i7e27168_app9.pdf]

Data Recorded	Intervention Group		Control Group
	Registered through OW	Registered Independently	
Number of FSW reached through OW at least 6 months since their last HIV test	Yes	No	Yes
Numbers of FSW offered for HIV test at health facility	Yes	No	Yes
Numbers agreeing and disagreeing to testing at a health facility	Yes	No	Yes
Of those agreeing to getting tested at a health facility, the numbers that actually went to a health facility and were tested for HIV	Yes	No	Yes
Number of FSW accessing website independently	No	Yes	No
Number of FSW offered for OFT screening	Yes	No	No
Number of FSW eligible for CBS	Yes	Yes	No
Number of FSW receiving HIVST	Yes	Yes	No
Number of eligible FSW receiving blood test	Yes	No	No
Number of FSW screened	Yes	Yes	No
Number of reactive HIVST	Yes	Yes	No
Number of confirmatory tests among FSW with reactive HIVST results	Yes	Yes	No
Number of FSW initiating ART	Yes	Yes	Yes
