# Supplementary material for: Web-Based Multifaceted Approach for Community-Based HIV Self-Testing Among Female Sex Workers in Indonesia: Protocol for a Randomized Community Trial
Source: JMIR Res Protoc. 2021 Jul 21;10(7):e27168. doi: 10.2196/27168 (PMC8339988; doi:10.2196/27168)
Supplement: Multimedia Appendix 10 [file resprot_v10i7e27168_app10.pdf]

Primary Outcomes	Description
HIV testing uptake	Proportion of those who receive facility-based HIV testing, including post-OFT confirmatory tests
HIV positivity rate	Proportion of those who receive both OFT and facility-based HIV testing
ART initiation rate	Proportion of those confirmed to be HIV positive who initiate ART

Secondary Outcomes	Description
**Characteristics (baseline survey)**	
Demographics	Age, education, marital status, place of recruitment
Sexual behavior	Age of first sexual transaction, condom use, number of clients in the past 7 days, stable partner
History of HIV test	Ever undergone HIV testing, last HIV testing, ever offered HIV testing within the past year, whether or not accepted HIV testing, reason for not accepting HIV testing
History of sexually transmissible infections	History of discomfort during sex or around genitalia, ever diagnosed with STI by a doctor within the past six months
Client base	Internet or social media, *lokalisasi*, karaoke, bar, massage parlor, friends, etc.
Knowledge of OFT	Prior knowledge of OFT and perception of OFT promotion
Stigma score	Bahasa Indonesia version of 12-item short HIV stigma scale
**Perception on OFT (OFT post-test survey)**	
Clarity of OFT information	Scale of 1-5, 1 being very unclear and 5 being very clear
Peer or OW assistance	Yes/No
Difficulty of use	Scale of 1-5, 1 being very difficult and 5 being very easy
Difficulty of interpreting results	Scale of 1-5, 1 being very difficult and 5 being very easy
Perceived support (only for assisted OFT)	Scale of 1-5, 1 being very unsupportive and 5 being very supportive
Willingness to receive routine testing after OFT (only for negative OFT results)	Scale of 1-5, 1 being very unwilling and 5 being very willing
Experience of testing	Scale of 1-5, 1 being very unpleasant and 5 being very pleasant
Certainty of result	Scale of 1-5, 1 being very uncertain and 5 being very certain
OFT result	Positive, negative, or indeterminate
Desire to confirm OFT result	Scale of 1-5, 1 being very weak desire and 5 being very strong desire
Desire to recommend OFT testing to peers	Scale of 1-5, 1 being very weak desire and 5 being very strong desire
Reason for lack of or wear desire to receive facility-based testing	Multiple answers with free-text option
Suggestions for OFT	Multiple answers with free-text option
Comments to improve study procedure	Multiple answers with free-text option
